# *Perlodinellashennongjia* sp. nov., a new species of *Perlodinella* Klapálek (Plecoptera, Perlodidae) from the central area of China

**DOI:** 10.3897/BDJ.10.e87247

**Published:** 2022-08-29

**Authors:** Zhi-Teng Chen, Yi-Yang Xu, Zi-Hao Shen

**Affiliations:** 1 Jiangsu University of Science and Technology, Zhenjiang, China Jiangsu University of Science and Technology Zhenjiang China; 2 Hubei Broad Nature Technology Service Co., Ltd., Wuhan, China Hubei Broad Nature Technology Service Co., Ltd. Wuhan China

**Keywords:** Plecoptera, Perlodidae, *Perlodinella*, new species, stonefly

## Abstract

**Background:**

*Perlodinella* Klapálek, 1912 is a small stonefly genus in the Palearctic areas of China and its biodiversity is underestimated.

**New information:**

This paper reports a new species of *Perlodinella*, *Perlodinellashennongjia* sp. nov. in the Dajiuhu National Wetland Park of Shennongjia Forestry District, Hubei Province, central China. The description and illustrations of the new species are provided, based on male adults, female adults and eggs. The new species can be distinguished from its congeners by the characters of male and female genitalia and the egg structure.

## Introduction

Stoneflies (Plecoptera) are known as a relatively small group of hemi-metabolous aquatic insects. The stonefly genus *Perlodinella* Klapálek, 1912 belongs to Perlodidae and is distributed in the Palearctic areas of China (Klapálek 1912, Dewalt et al. 2022). Currently, *Perlodinella* includes nine species all of which are in China: *P.kozlovi* Klapálek, 1912, *P.unimacula* Klapálek, 1912, *P.microlobata* Wu, 1938a, *P.apicalis* Kimmins, 1947, *P.fuliginosa* Wu, 1973, *P.tatunga* Wu, 1973, *P.mazehaoi* Chen, 2019, *P.tibetensis* Huo & Du, 2022 and the recently transferred *P.epiproctalis* (Zwick, 1997) from the genus *Rauserodes* Zwick, 1999 (Klapálek 1912, Wu 1938a, Kimmins 1947, Wu 1973, Zwick 1997, Chen 2019, Huo et al. 2022). However, most older species included in *Perlodinella* lack detailed descriptions and clear photos of male genitalia and egg morphology. For instance, *P.unimacula* is known only by female characters and, thus, its comparison with congeners is restricted to the female morphology. The discovery of more species and a detailed study of their morphology are essential to better understand the generic definition and biodiversity of *Perlodinella*.

In the present paper, a new species of the genus *Perlodinella* from the Dajiuhu National Wetland Park of Shennongjia Forestry District, Hubei Province, central China, is described and illustrated, based on both sexes and eggs.

## Materials and methods

The specimens were collected on the waterside cement road from Dajiuhu National Wetland Park in Shennongjia Forestry District, Hubei Province, central China. The abdomen of the male holotype was soaked in 10% sodium hydroxide (NaOH) for half an hour to extrude the aedeagus and eversible paraproct lobes. Immature eggs were taken from inside of the female abdomen. Observations and measurements were performed with a SDPTOP SZM45 stereomicroscope. Colour images were taken using a Canon EOS 6D digital camera with a Canon MP-E 65 mm 5X macro lens. All images were optimised and assembled into plates with Adobe Photoshop CS6. The holotype and paratypes are deposited in the Insect Collection of Jiangsu University of Science and Technology, Jiangsu Province, China (ICJUST). The terminology of wing venation follows that of Béthoux (2005). The following abbreviations are used: C, costa; Sc, subcosta; RA, anterior radius; RP, posterior radius.

## Taxon treatments

### Perlodinella shennongjia

Chen, Xu & Shen, 2022 sp. n.

A82179F1-AE25-551F-8966-02877E7BF06B

0DB574AB-1770-4CB3-81AB-FC4BE4A3C26B

#### Materials

- **Type status:** Holotype. **Occurrence:** individualCount: 1; sex: male; **Taxon:** kingdom: Animalia; phylum: Arthropoda; class: Insecta; order: Plecoptera; family: Perlodidae; genus: Filchneria; specificEpithet: *shennongjia*; taxonRank: species; nomenclaturalCode: ICZN; **Location:** country: China; stateProvince: Hubei; municipality: Shennongjia Forestry District; locality: Dajiuhu National Wetland Park; verbatimElevation: 1551 m; verbatimCoordinates: 110°7′33.9″N, 31°27′5″E; **Identification:** identifiedBy: Zhi-Teng Chen; **Event:** verbatimEventDate: 31-03-2022; **Record Level:** institutionCode: ICJUST
- **Type status:** Paratype. **Occurrence:** individualCount: 11; sex: 7 males, 4 females; **Taxon:** kingdom: Animalia; phylum: Arthropoda; class: Insecta; order: Plecoptera; family: Perlodidae; genus: Filchneria; specificEpithet: *shennongjia*; taxonRank: species; nomenclaturalCode: ICZN; **Location:** country: China; stateProvince: Hubei; municipality: Shennongjia Forestry District; locality: Dajiuhu National Wetland Park; verbatimElevation: 1551 m; verbatimCoordinates: 110°7′33.9″N, 31°27′5″E; **Identification:** identifiedBy: Zhi-Teng Chen; **Event:** verbatimEventDate: 31-03-2022; **Record Level:** institutionCode: ICJUST

#### Description

##### Male

**Figure 1.**
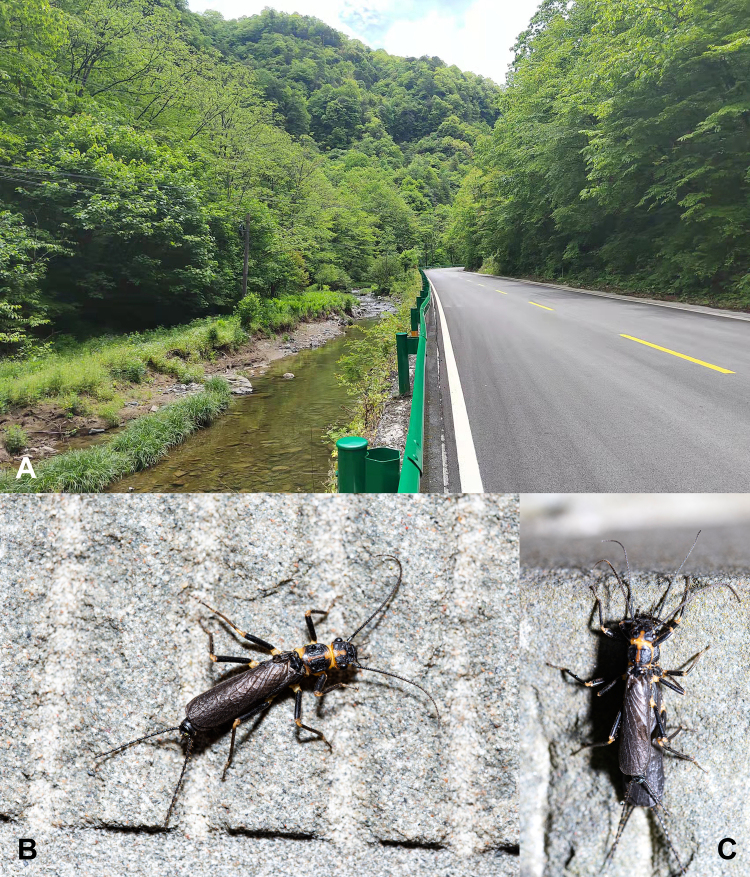
*Perlodinellashennongjia* sp. nov. **A** Habitat in Dajiuhu National Wetland Park, Shennongjia Forestry District, Hubei Province, China, photo by Yi-Yang Xu; **B** Living habitus of male, photo by Yi-Yang Xu; **C** Living habitus of a pair of mating adults, photo by Yi-Yang Xu.

**Figure 2.**
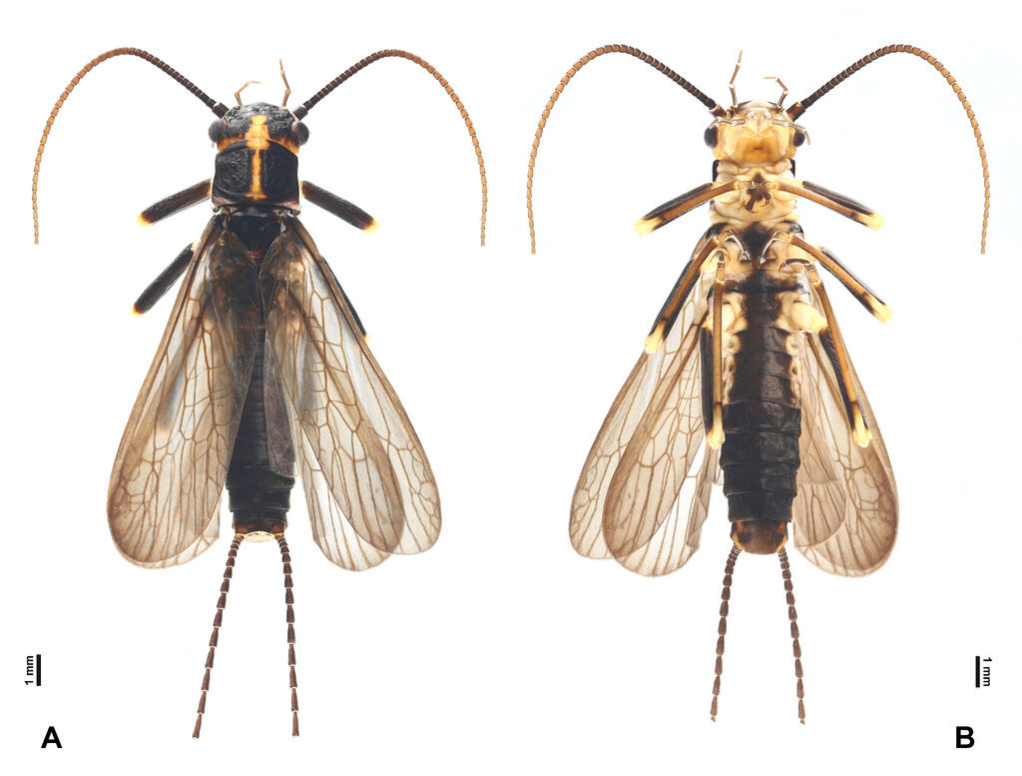
*Perlodinellashennongjia* sp. nov., male holotype **A** Habitus, dorsal view; **B** Habitus, ventral view.

Body length (from anterior of head to posterior of paraprocts) 13.0-15.0 mm (examined specimen number = 8), living male near habitat stream and male in ethanol both dark brown (Figs 1, 2).

Head mostly dark brown dorsally, pale ventrally (Fig. 2); triocellate, anterior ocellus much smaller than posterior ocelli, ocellar area and posterior margin of head pale. Compound eyes dark and rounded. Antenna slender, length slightly longer than abdomen, all segments dark brown.

**Figure 3.**
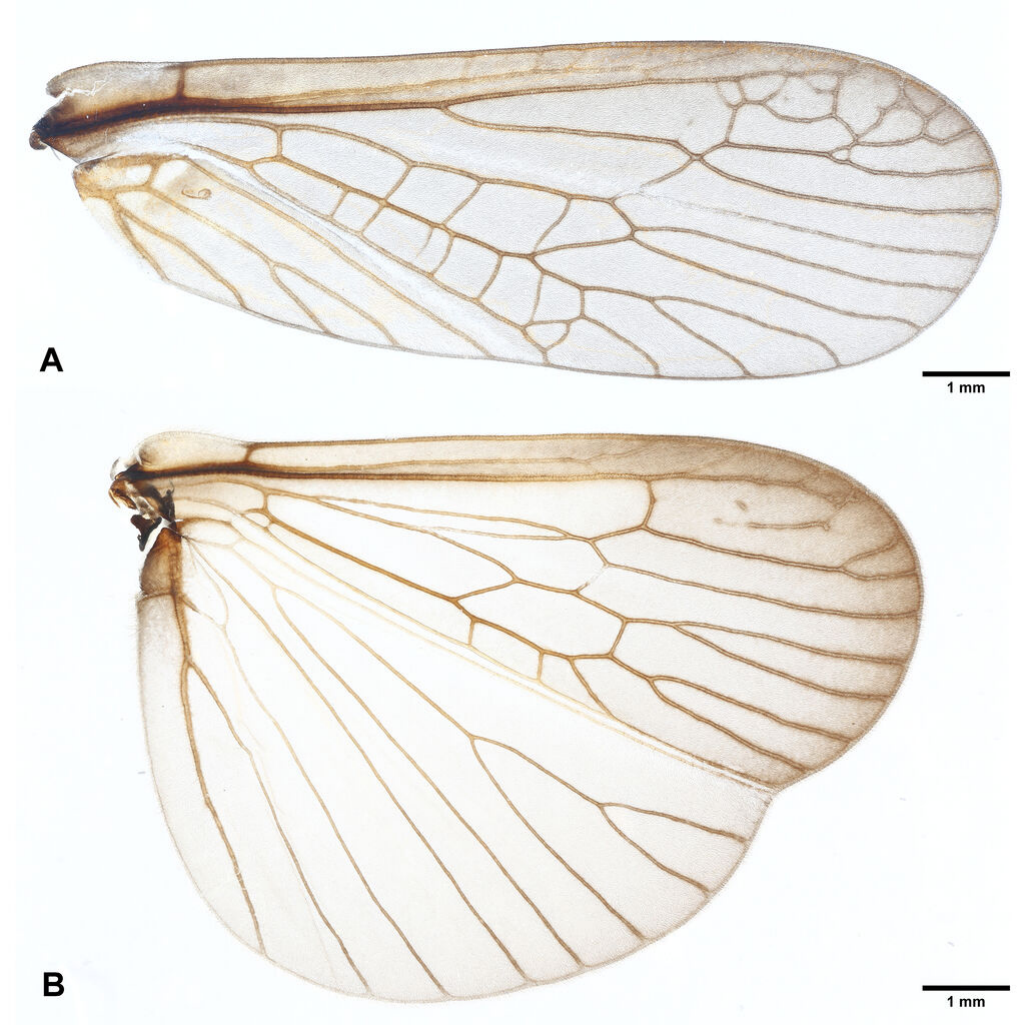
*Perlodinellashennongjia* sp. nov., male holotype **A** Right forewing, dorsal view; **B** Right hind-wing, dorsal view.

Pronotum subquadrate (Fig. 2), lateral margins nearly parallel, pigmentation dark brown, except pale median stripe, surface with several obscure rugosities. Prosternum mostly pale, medially with a fusiform, dark spot. Mesothoracic furcasternum branches reaching posterior of furcal pits. Basisterna and furcasterna of meso- and metathorax dark brown. Wings fully developed or slightly shortened (Figs 1, 2, 3), fore-wings length 10.0-12.0 mm (examined specimen number = 8), hind-wings length 8.0-10.0 mm (examined specimen number = 8); wing membrane subhyaline, veins brown. In fore-wings, apex with small net-like venation formed by apical branches of RA and RP; six to seven cross-veins present between C and Sc; anal area with four main anal veins. In hind-wings, apical net similar to that of fore-wing; anal area large and folded, with about ten anal branches. Leg background dark brown (Fig. 2); coxae, trochanters and joints between femora and tibiae pale; two giant tibial spurs present apically; claws slender and sharp.

**Figure 4.**
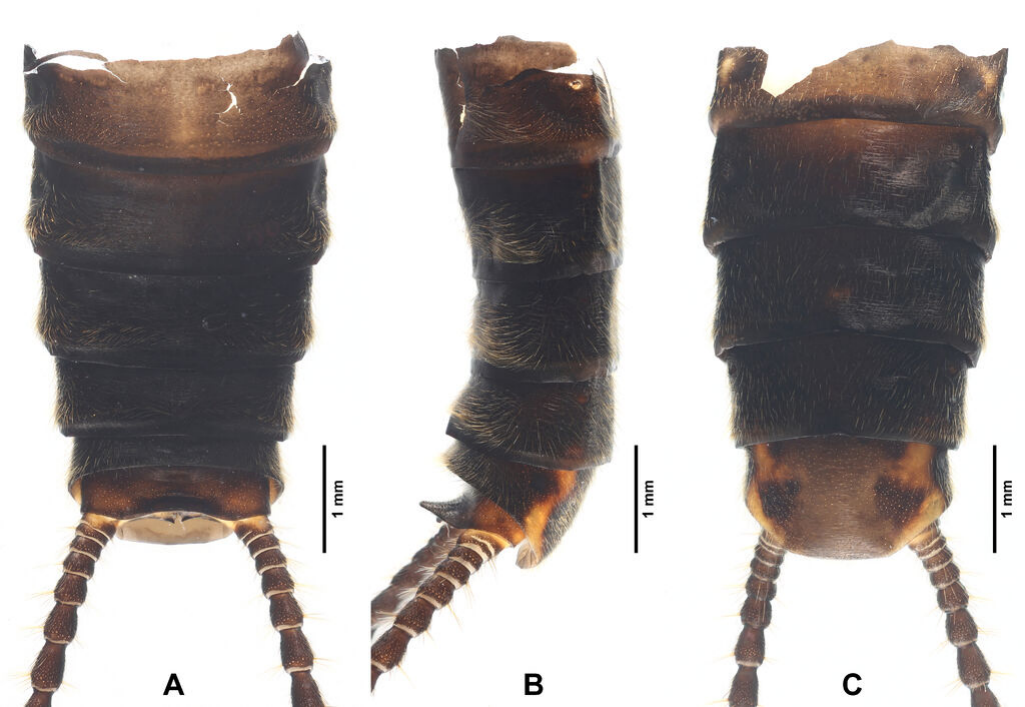
*Perlodinellashennongjia* sp. nov., male holotype **A** Abdomen, dorsal view; **B** Abdomen, lateral view; **C** Abdomen, ventral view.

**Figure 5.**
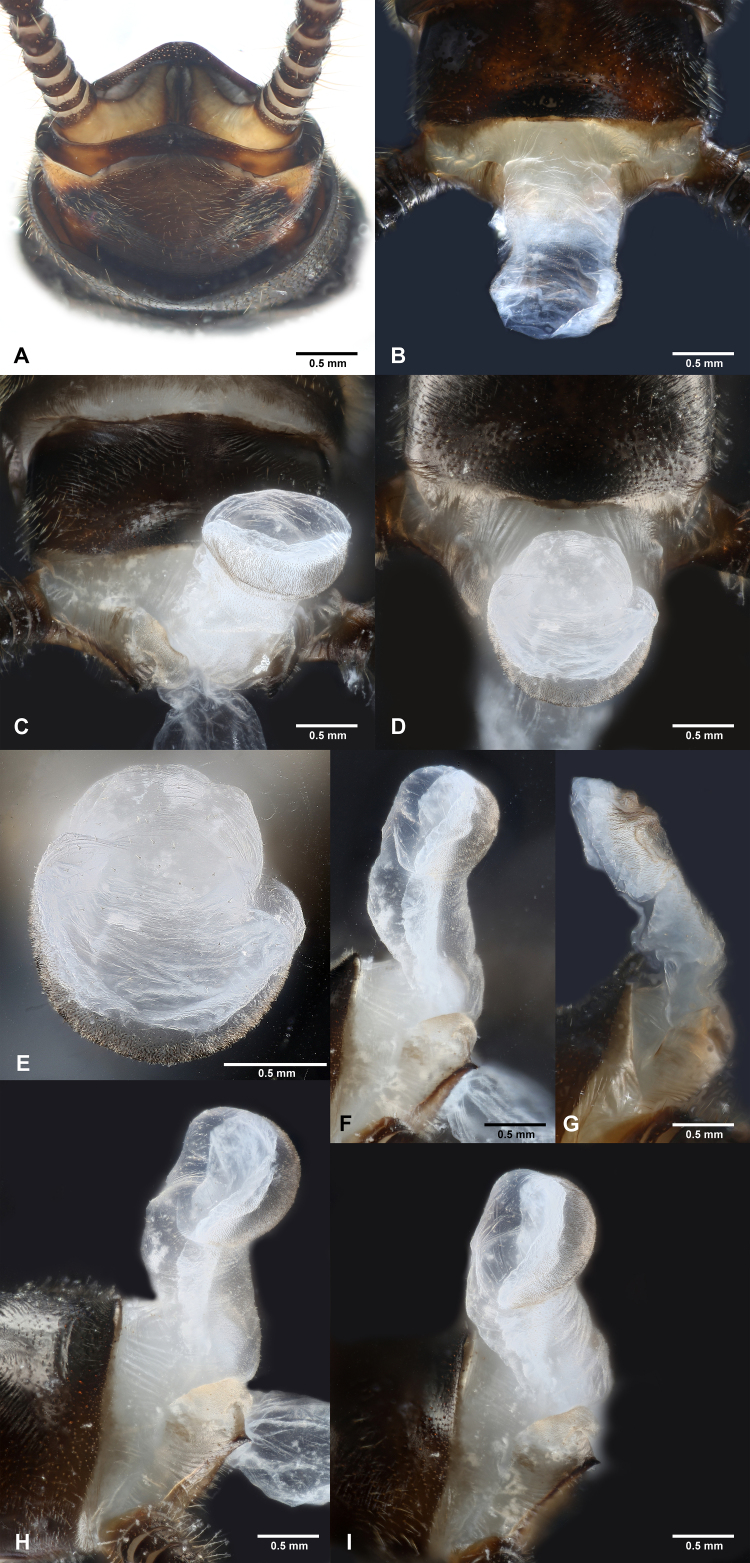
*Perlodinellashennongjia* sp. nov., male holotype **A** Male terminalia, caudal view; **B–D** Male terminalia, dorsal view; **E** Apex of aedeagus, dorsal view; **F–G** Aedeagus, lateral view; **H–I** Aedeagus, dorsolateral view.

Abdominal segments mostly dark (Figs 2, 4, 5), segments 1-4 divided into distinct terga and sterna by pale lateral membrane, pale lateral areas extending to sterna 5-6 in a paratype. Sternum 1 completely fused with metathorax. Sternum 9 elongated, mostly or entirely dark brown. Terga 6-9 not elevated at posterior half, with dense posterolateral hair patches. Posterior half of terga 8-10 with scattered sensilla basiconica. Tergum 10 strongly elevated, dorsally covered with dense short spines and sparse sensilla basiconica (Figs 4, 5); apex blunt, ventrally with scattered sensilla basiconica. Epiproct completely membranous (Fig. 5), thumb-shaped, basal half cylindrical, slightly constricted near mid-point, apical half rounded; dorsal surface covered with sparse patch of conical, dark spines, ventral surface covered with dense patch of conical, dark spines, apex scattered with sparse, conical, pale spines. Paraproct sclerite wide and resembling a parallelogram basally (Fig. 5), then gradually tapering into an inwardly pointed apex; anterior margins of paraproct sclerites dark; inner margins dark, not connected basally, nearly parallel at basal half, apical half of paraproct sclerites surrounding a circular median area. Paraproct lobe short, near bulbous, covered with dense, conical, pale spines and with several scattered, conical, dark spines. Cerci subequal in length to abdomen; each segment mostly dark brown, except paler basal ends, with a whorl of long bristles around distal end.

##### Female

**Figure 6.**
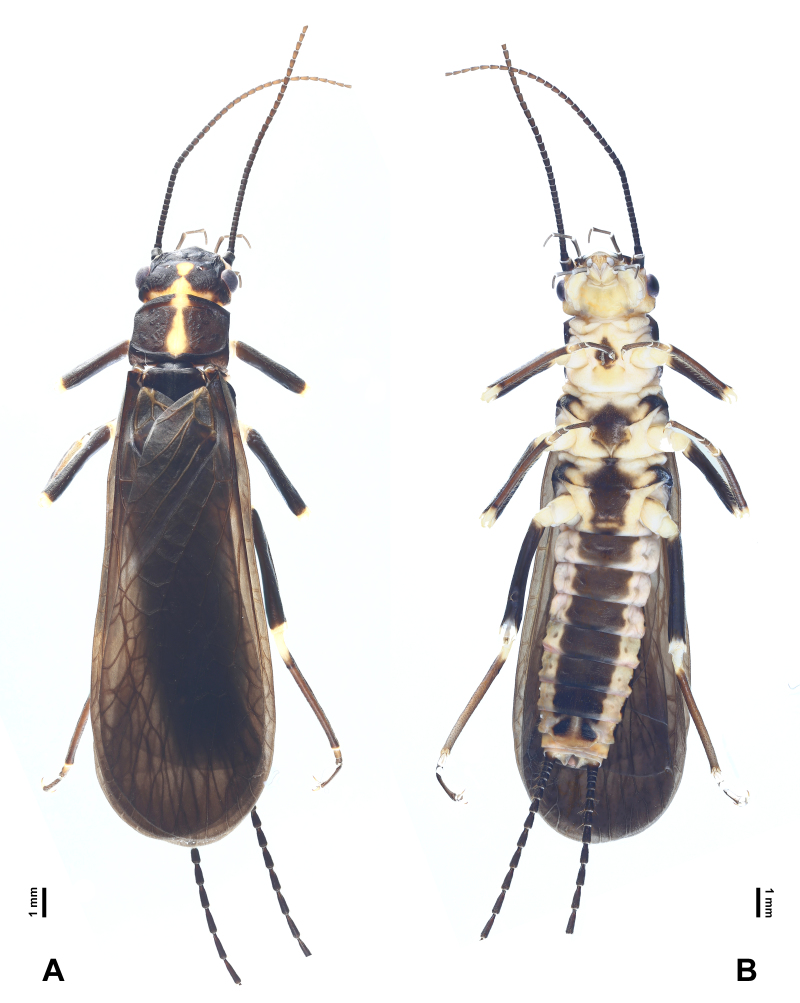
*Perlodinellashennongjia* sp. nov., female paratype **A** Female habitus, dorsal view; **B** Female habitus, ventral view.

Body length 17.0-19.0 mm (examined specimen number = 4), mostly dark brown (Fig. 6). Colour pattern similar to males.

**Figure 7.**
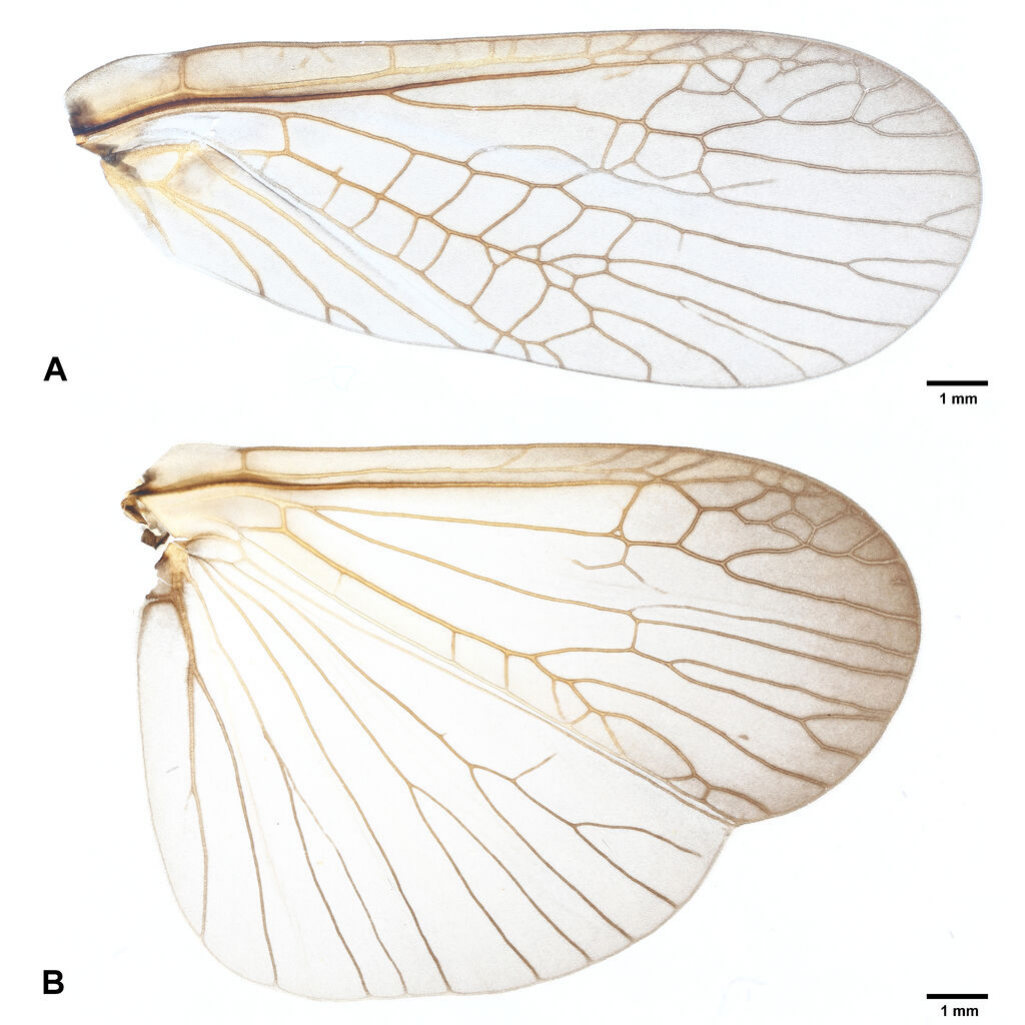
*Perlodinellashennongjia* sp. nov., female paratype **A** Right fore-wing, dorsal view; **B** Right hind-wing, dorsal view.

Macropterous (Figs 6, 7); fore-wings length 15.0-16.0 mm, hind-wings length 13.0-14.0 mm (examined specimen number = 4); wing membrane subhyaline, veins brown. Wing venation similar to males.

Abdomen dorsally dark brown; abdominal sterna 1-7 with a continuous dark brown, median stripe, lateral areas pale (Fig. 6). Abdominal sternum 8 with four subtriangular dark sclerites, anterior ones smaller, posterior ones larger, median line pale. Subgenital plate broad, posterior margin rounded, nearly reaching posterior margin of sternum 9. Sterna 9-10 pale and short. Paraproct subtriangular, apex blunt, with grey inner and posterior margins.

##### Egg

**Figure 8.**
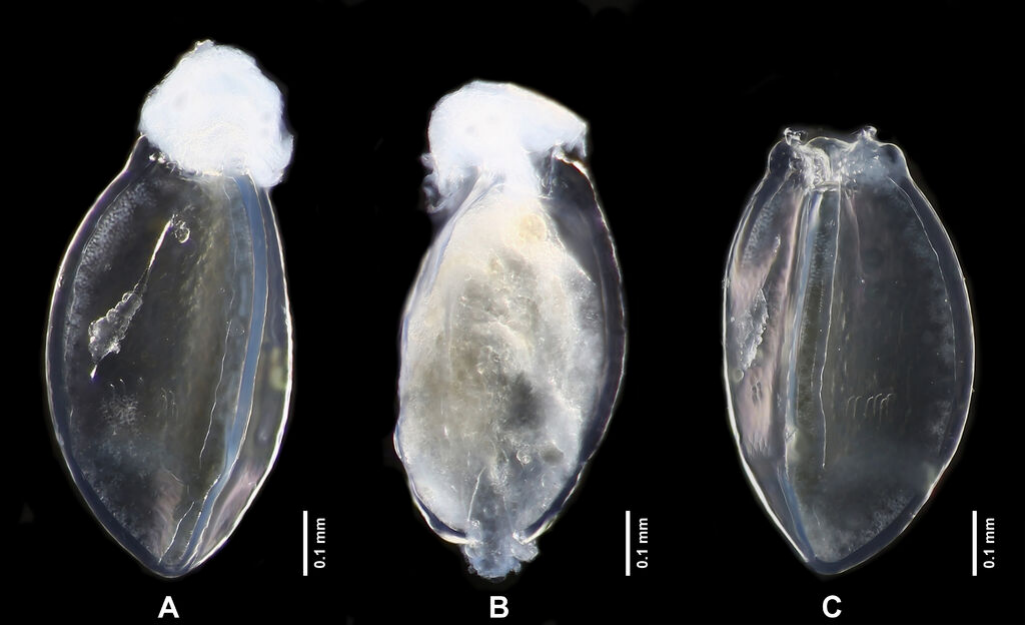
*Perlodinellashennongjia* sp. nov., immature eggs **A** Egg with anchor preserved, lateral view; **B** Less-developed egg, lateral view; **C** Egg with anchor removed, lateral view.

Length ca. 800 μm; width ca. 400 μm. Trilateral (Fig. 8), with three conspicuous longitudinal ridges. Each side of egg with a transverse ridge near posterior pole. Anterior area of each transverse ridge with one row of several micropyles. Chorion relatively smooth. Anchor completely membranous, mushroom-shaped in lateral view, surface covered with dense granules. Collar sessile and short, with sinuous anterior margins.

#### Diagnosis

The new species is diagnostic by the following combination of features: mesothoracic furcasternum branches reaching posterior of furcal pits; hind-wings with broad anal area; male abdominal segments 1-4 divided into distinct terga and sterna; terga 6-9 not elevated at posterior half, with dense posterolateral hair patches; terga 8-10 with scattered sensilla basiconica on posterior half; tergum 10 strongly elevated, dorsally covered with dense short spines and sparse sensilla basiconica, apex blunt, ventrally with scattered sensilla basiconica; aedeagus membranous, thumb-shaped, dorsally covered with sparse dark spines, ventrally covered with dense dark spines, apex with sparse pale spines; paraproct sclerite basally resembling a parallelogram, apex inwardly pointed, anterior and inner margins dark, not connected basally; eversible paraproct lobe short, near bulbous, covered with dense pale spines and several scattered dark spines; female abdominal sterna 1-7 with a continuous dark median stripe, sternum 8 with four subtriangular dark sclerites, sterna 9-10 pale and short; subgenital plate broad, elongated and rounded; eggs trilateral, with both longitudinal and transverse ridges, micropyles present.

*Perlodinellashennongjia* sp. nov. can be easily distinguished from *P.kozlovi* and *P.epiproctalis* by the unlobed epiproct (Zwick 1997, Huo et al. 2022), from *P.unimacula*, *P.microlobata*, *P.fuliginosa* and *P.tatunga* by the rounded female subgenital plate without any notch or lobes (Wu 1938a, Wu 1938b, Wu 1973), from *P.apicalis* by the absence of dark brown femora and downcurved hook on paraproct (Kimmins 1947), from *P.mazehaoi* by the distinctly shorter male tergum 10 and different shape of female subgenital plate (Chen 2019) and from *P.tibetensis* by the paraproct sclerites pointing inwards instead of outwards (Huo et al. 2022).

#### Etymology

The new species is named after its type locality, the Shennongjia Forestry District.

#### Distribution

The new species is currently only known from the Shennongjia Forestry District, Hubei Province, China.

## Supplementary Material

XML Treatment for Perlodinella shennongjia
